# Impact of methods used to express levels of circulating fatty acids on the degree and direction of associations with blood lipids in humans

**DOI:** 10.1017/S0007114515004341

**Published:** 2016-01-28

**Authors:** Susan Sergeant, Ingo Ruczinski, Priscilla Ivester, Tammy C. Lee, Timothy M. Morgan, Barbara J. Nicklas, Rasika A. Mathias, Floyd H. Chilton

**Affiliations:** 1 Center for Botanical Lipids and Inflammatory Disease Prevention, Wake Forest School of Medicine, Medical Center Blvd, Winston-Salem, NC 27157, USA; 2 Department of Biochemistry, Wake Forest School of Medicine, Medical Center Blvd, Winston-Salem, NC 27157, USA; 3 Department of Biostatistics, Bloomberg School of Public Health, Johns Hopkins University, Baltimore, MD 21205, USA; 4 Department of Physiology/Pharmacology, Wake Forest School of Medicine, Medical Center Blvd, Winston-Salem, NC 27157, USA; 5 Department of Public Health Sciences/Biostatistical Sciences, Wake Forest School of Medicine, Medical Center Blvd, Winston-Salem, NC 27157, USA; 6 Department of Internal Medicine/Gerontology and Geriatric Medicine, Wake Forest School of Medicine, Medical Center Blvd, Winston-Salem, NC 27157, USA; 7 Department of Medicine, Division of Allergy and Clinical Immunology, The Johns Hopkins University, Baltimore, MD 21224, USA; 8 Department of Molecular Medicine and Translational Sciences, Wake Forest School of Medicine, Medical Center Blvd, Winston-Salem, NC 27157, USA

**Keywords:** PUFA, Lipid biomarkers, Linoleic acid, Arachidonic acid

## Abstract

Numerous studies have examined relationships between disease biomarkers (such as blood lipids) and levels of circulating or cellular fatty acids. In such association studies, fatty acids have typically been expressed as the percentage of a particular fatty acid relative to the total fatty acids in a sample. Using two human cohorts, this study examined relationships between blood lipids (TAG, and LDL, HDL or total cholesterol) and circulating fatty acids expressed either as a percentage of total or as concentration in serum. The direction of the correlation between stearic acid, linoleic acid, dihomo-*γ*-linolenic acid, arachidonic acid and DHA and circulating TAG reversed when fatty acids were expressed as concentrations *v*. a percentage of total. Similar reversals were observed for these fatty acids when examining their associations with the ratio of total cholesterol:HDL-cholesterol. This reversal pattern was replicated in serum samples from both human cohorts. The correlations between blood lipids and fatty acids expressed as a percentage of total could be mathematically modelled from the concentration data. These data reveal that the different methods of expressing fatty acids lead to dissimilar correlations between blood lipids and certain fatty acids. This study raises important questions about how such reversals in association patterns impact the interpretation of numerous association studies evaluating fatty acids and their relationships with disease biomarkers or risk.

Fatty acids have diverse biological roles that are central to human health and disease. Qualitative and quantitative changes in the dietary fat content as a result of the modern Western diet^(^ ^1^ ^–^ ^3^ ^)^ have altered the balance of circulating and resultant tissue levels of SFA, MUFA and PUFA and their metabolites. Many of these changes have been associated with alterations in the levels of disease biomarkers and chronic inflammation, which may impact the risk for diseases such as CVD, diabetes and cancer^(^ ^4^ ^–^ ^8^ ^)^.

In mammals, SFA and MUFA can be obtained preformed from the diet or synthesised *in vivo*. In contrast, PUFA must be obtained from the diet. The two essential dietary PUFA precursors are the eighteen-carbon *n*-6 PUFA, linoleic acid (LA, C18 : 2*n*-6), and the *n*-3 PUFA, *α*-linolenic acid (ALA, C18 : 3*n*-3). The long-chain PUFA can be obtained from the diet or biochemically derived from essential PUFA through a series of alternating desaturation and elongation enzymatic steps^(^ ^9^ ^)^. For example, the *n*-6 long-chain PUFA, arachidonic acid (ARA, C20 : 4*n*-6), can be synthesised from LA; the *n*-3 long-chain PUFA, EPA (C20 : 5*n*-3) and DPA (C22 : 5*n*-3), can be synthesised from ALA.

There remain important questions concerning the recommendations for quantities and ratios of dietary fatty acids needed to maintain health. In particular, there has been an intense debate as to whether replacing dietary SFA with the *n*-6 PUFA LA, abundant in vegetable oils, has benefited or harmed human health^(^ ^10^ ^,^ ^11^ ^)^. The question of dietary requirements has been complicated by recent studies illustrating wide genetic variations in the capacity of individuals and racial/ethnic groups to metabolise dietary PUFA^(^ ^11^ ^–^ ^13^ ^)^.

Consequently numerous studies have directly examined the relationships between levels of fatty acids in circulation or cells/tissues and disease biomarkers and the incidence of human disease^(^ ^14^ ^–^ ^23^ ^)^. In such studies, fatty acid levels have been evaluated in whole blood, erythrocytes, serum, plasma or fractionated plasma components (phospholipids, cholesterol esters, TAG, unesterified fatty acids) utilising GC with flame ionisation detection. Fatty acid data from such studies typically have been expressed as a percentage of an individual fatty acid normalised to the total amount of all fatty acids measured in the sample (i.e. 100 %). This expression method can serve to overcome inter-study variations in extraction and separation efficiencies in fatty acid analyses. In contrast, a few studies have reported fatty acid levels as absolute concentrations^(^ ^24^ ^,^ ^25^ ^)^. This is interesting as other blood lipids and biomarkers are generally reported in units of concentration.

The current study examined associations between levels of circulating total fatty acids and blood lipids (TAG, and LDL, HDL or total cholesterol (TC)) in two human cohorts. This study has identified strong associations between circulating fatty acids and lipid biomarkers. However, these data also point out striking differences in associations garnered from data utilising the common practice of expressing fatty acids as a percentage of total *v*. data in which fatty acids are expressed as in terms of concentration (mg/dl or mmol/l).

## Methods

### Participants

The Diet, Exercise, Metabolism and Obesity in older women (DEMO) study was conducted from 2003 to 2007; the details of this study have been described elsewhere^(^ ^26^ ^)^. The DEMO cohort consisted of ninety-three women who were overweight, but were otherwise healthy. A second, replicate, cohort was from a study designed to assess the effects of botanical and fish oils on disease biomarkers in the diabetes/metabolic syndrome subjects that was carried out from 2012 to 2013^(^ ^27^ ^)^. The latter diabetes/metabolic syndrome cohort was composed of fifty-nine participants (59 % women) who had been diagnosed with either early-stage type 2 diabetes or had the metabolic syndrome^(^ ^28^ ^,^ ^29^ ^)^. Both studies were approved by the Wake Forest School of Medicine Institutional Review Board, and all participants gave written, informed consent for their respective study as well as for future research use of their archived biospecimens. Blood lipid profiles (including TAG, and LDL, HDL or TC) were measured in plasma (DEMO) or serum (the diabetes/metabolic syndrome study) at baseline (pre-intervention) by a qualified clinical laboratory (LabCorp).

### Fatty acid analysis

Total serum fatty acids were analysed by GC with flame ionisation detection^(^ ^30^ ^)^ using a Hewlett Packard 5890 instrument (Agilent) with an Agilent J&W DB-23 column (30 m, 0·25 mm ID, 0·25 µm film; Agilent) fitted with an inert pre-column (1 m, 0·53 mm ID) for cool on-column injection. Fatty acid was cleaved from complex lipids and converted to methyl esters in duplicate serum samples (100 μl) utilising a modification of Metcalfe *et al.* ^(^ ^31^ ^)^. Fatty acids in samples were identified on the basis of retention times of commercially available authentic fatty acid methyl ester standards. Triheptadecanoin (100 µg; TAG of C17 : 0; Nu-Chek Prep) was included in the samples as an internal standard. Fatty acids (23–29 peaks) were routinely identified and these accounted for >99 % of the total fatty acids in the sample. Archival baseline serum samples from the DEMO study had been stored at −80°C for 6–10 years. The stability of circulating fatty acids over this time period has been reported to be excellent^(^ ^32^ ^)^. Fatty acid data are presented as the percentage of total fatty acids in the sample or expressed as concentration (mmol/l) in serum. Data for nineteen of the most abundant fatty acids were used for association analyses.

### Statistical analyses

Measures of association between selected serum fatty acids and blood lipids are reported as Pearson’s sample correlation coefficients, with statistical significance for non-zero correlations derived from hypothesis tests using Fisher’s *z*-transformation. Theoretical predictions for the correlations between circulating lipids and fatty acid expressed as a percentage of total were derived using standard linear model theory and a first-order Taylor expansion for functions of multivariate random variables^(^ ^33^ ^)^, described in detail in the online Supplementary Materials. All analyses were carried out using the statistical environment R (http://cran.r-project.org).

## Results

### Characteristics of the study populations

The DEMO cohort consisted of ninety-three women (61 % European-American, 39 % African-American) ranging in age from 50 to 65 years, with an average age of 56·9 (sd 4·4) years. Aside from being overweight (BMI, 33·3 (sd 3·8) kg/m^2^; range 26–41·3), this cohort of postmenopausal women was generally healthy, as diabetes, coronary artery disease, cancer, diseases of the liver, kidneys or lungs and tobacco use were criteria for exclusion from the study. In addition, the individuals in the cohort did not, on average, meet the criteria to be classified as having the metabolic syndrome^(^ ^28^ ^,^ ^29^ ^)^.

A replication cohort consisted of subjects (*n* 59; 59 % European-American, 39 % African-American, 2 % Asian) with the diabetes/metabolic syndrome and was composed of thirty-five women (59 %) and twenty-four men. The average age of this cohort was 58·1 (sd 5·6) years (range 40–74) and the average BMI was 34·2 (sd 5·6) kg/m^2^ (range 23–49).

### Circulating fatty acid profile

Total fatty acids were analysed in baseline fasting serum samples (before any intervention) of the ninety-three women from the DEMO cohort. A total of fifty-nine serum samples were available for analysis from the diabetes/metabolic syndrome cohort. Table 1 shows the fatty acid profile of the twenty-three to twenty-nine fatty acids routinely detected in these serum samples. These fatty acids accounted for >99 % of the fatty acids in the samples. The fatty acid data are presented using two methods of expression: the absolute concentration in μmol/l of serum and the relative amount to total fatty acids (i.e. percentage of total fatty acid) in serum. Nineteen of the most abundant circulating fatty acids were subsequently used for association analyses.

**Table 1:** Fatty acid profile of the study populations* (Mean values and standard deviations)

		DEMO cohort				Diabetes cohort			
		µmol/l		Total (%)		µmol/l		Total (%)	
Common names		Mean	sd	Mean	sd	Mean	sd	Mean	sd
	SFA								
Myristic acid	C14 : 0	99·9	53·5	0·8	0·3	111·7	66·3	0·9	0·4
	C15 : 0	23·7	9·9	0·2	0·1	21·2	14·7	0·2	0·1
Palmitic acid	C16 : 0	2654·7	735·2	21·5	1·6	2895·1	906·3	22·9	2·0
Stearic acid	C18 : 0	891·8	202·8	7·3	0·8	884·1	226·9	7·1	0·7
Arachidic acid	C20 : 0	1·0	3·3	<0·1	<0·1	0·6	2·9	<0·1	<0·1
	MUFA								
Myristoleic acid	C14 : 1	1·5	7·4	<0·1	0·1	<0·4	1·3	<0·1	<0·1
Palmitoleic acid	C16 : 1*n*-7	261·6	153·1	2·1	0·8	238·6	121·9	1·9	0·7
	C17 : 1	21·8	13·4	0·2	0·1	13·8	16·2	0·1	0·1
Elaidic acid	C18 : 1*n*-9*t*	58·0	49·8	0·5	0·4	20·7	33·9	0·2	0·3
Oleic acid	C18 : 1*n*-9	2392·8	763·4	19·3	2·3	2610·0	936·0	20·5	3·5
Vaccenic acid	C18 : 1*n*-7	206·4	64·7	1·7	0·3	201·7	65·1	1·6	0·3
	C20 : 1*n*-9	5·0	7·2	<0·1	<0·1	8·9	11·9	<0·1	<0·1
Erucic acid	C22 : 1	1·3	7·8	<0·1	<0·1	2·0	11·2	<0·1	<0·1
	*n*-6 PUFA								
Linolelaidic acid	C18 : 2*n*-6t	26·6	18·9	0·2	0·1	–	–	–	–
Linoleic acid	C18 : 2*n*-6	3825·4	769·3	31·7	3·9	3880·0	963·0	31·4	5·2
*γ*-Linolenic acid	C18 : 3*n*-6	68·8	34·4	0·5	0·2	70·2	28·9	0·6	0·2
	C20 : 2*n*-6	27·9	10·0	0·2	0·1	36·2	13·7	0·3	0·1
Dihomo-*γ*-linolenic acid	C20 : 3*n*-6	171·1	58·8	1·4	0·3	171·4	57·2	1·4	0·3
Arachidonic acid	C20 : 4*n*-6	971·5	220·1	8·1	1·8	962·7	223·3	7·9	1·9
Adrenic acid	C22 : 4*n*-6	25·6	7·6	0·2	0·1	–	–	–	–
	C22 : 5*n*-6	24·3	18·7	0·2	0·2	–	–	–	–
	*n*-3 PUFA								
*α*-Linolenic acid	C18 : 3*n*-3	88·3	37·7	0·7	0·2	96·8	46·3	0·8	0·3
Stearidonic acid	C18 : 4*n*-3	10·7	21·7	0·1	0·2	2·4	10·1	<0·1	0·1
	C20 : 3*n*-3	45·2	56·8	0·4	0·5	–	–	–	–
	C20 : 4*n*-3	1·3	4·9	<0·1	<0·1	**–**	**–**	**–**	**–**
EPA	C20 : 5*n*-3	77·8	52·8	0·6	0·4	61·8	32·1	0·5	0·2
DPA	C22 : 5*n*-3	52·0	16·6	0·4	0·1	58·2	18·7	0·5	0·1
DHA	C22 : 6*n*-3	191·6	69·4	1·6	0·6	175·2	58·4	1·4	0·4

DEMO, Diet, Exercise, Metabolism and Obesity in Older Women.

* Total fatty acids were measured in fasting serum as described in Methods. The data are presented as fatty acid concentration in units of µmol/l and as percentage of total fatty acids. The twenty-three to twenty-nine fatty acids shown account for >99 % of the eluted peaks.

### Relationship between selected serum fatty acids and circulating TAG levels

**Fig. 1:**
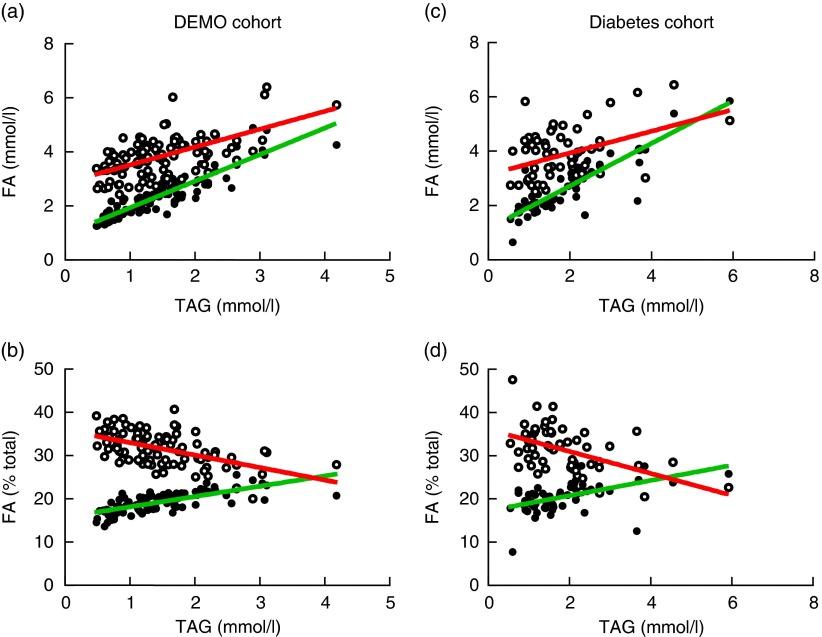
Impact of fatty acid (FA) expression method on the TAG relationship with oleic acid (OA, C18 : 1*n*-9) and linoleic acid (LA, C18 : 2*n*-6). The relationship between TAG and the most abundant circulating FA, LA (31 %), differs (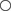, C18 : 2*n*-6) on the basis of the method of FA expression: concentration (mmol/l) (a); or as percentage of total (b) in the Diet, Exercise, Metabolism and Obesity in Older Women (DEMO) cohort. The same relationships were also examined in the replicate population (the diabetes/metabolic syndrome cohort; right panels) as concentration (mmol/l) (c); or as percentage of total (d). The relationship between TAG and OA (19–20 %) is unaffected (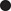, C18 : 1*n*-9) by the method of FA expression. For visualisation, the linear regression line is shown for each data set.

Fig. 1 shows the statistically significant associations between TAG and the two most abundant circulating fatty acids, LA (31 %) and oleic acid (OA) (19–20 %), calculated as a percentage of total and as concentrations. However, the direction of the relationship between TAG and LA was negative when expressed as a percentage of total (Fig. 1(b); *r* −0·53; *P*<0·0001), but positive when expressed as the serum concentration (mmol/l) of LA (Fig. 1(a); *r* 0·60; *P*<0·0001). In contrast, the association between TAG and OA was positive using either fatty acid expression method (*r* 0·71 with *P*<0·0001 for percentage of total, Fig. 1(b); and *r* 0·90 with *P*<0·0001 for absolute concentration, Fig. 1(a)).

Using an independent data set from the diabetes study cohort, we observed similar patterns (Fig. 1(c) and (d)) of relationships between LA and OA and TAG levels. Here too, the direction of the relationship between TAG and LA was negative when expressed as a percentage of total (Fig. 1(d); *r* −0·53; *P*<0·0001), but positive when expressed as the serum concentration (mmol/l) of LA (Fig. 1(c); *r* 0·37; *P*<0·005), and the association between TAG and OA was positive using either fatty acid expression method (*r* 0·62 with *P*<0·0001 for percentage of total, Fig. 1(d); and *r* 0·87 with *P*<0·0001 for concentration, Fig. 1(c)). We observed that the relationship between the alternative forms of fatty acid expression and TAG levels was consistent whether examining African-American and European-American subgroups or the entire cohort (data not shown).

**Fig. 2:**
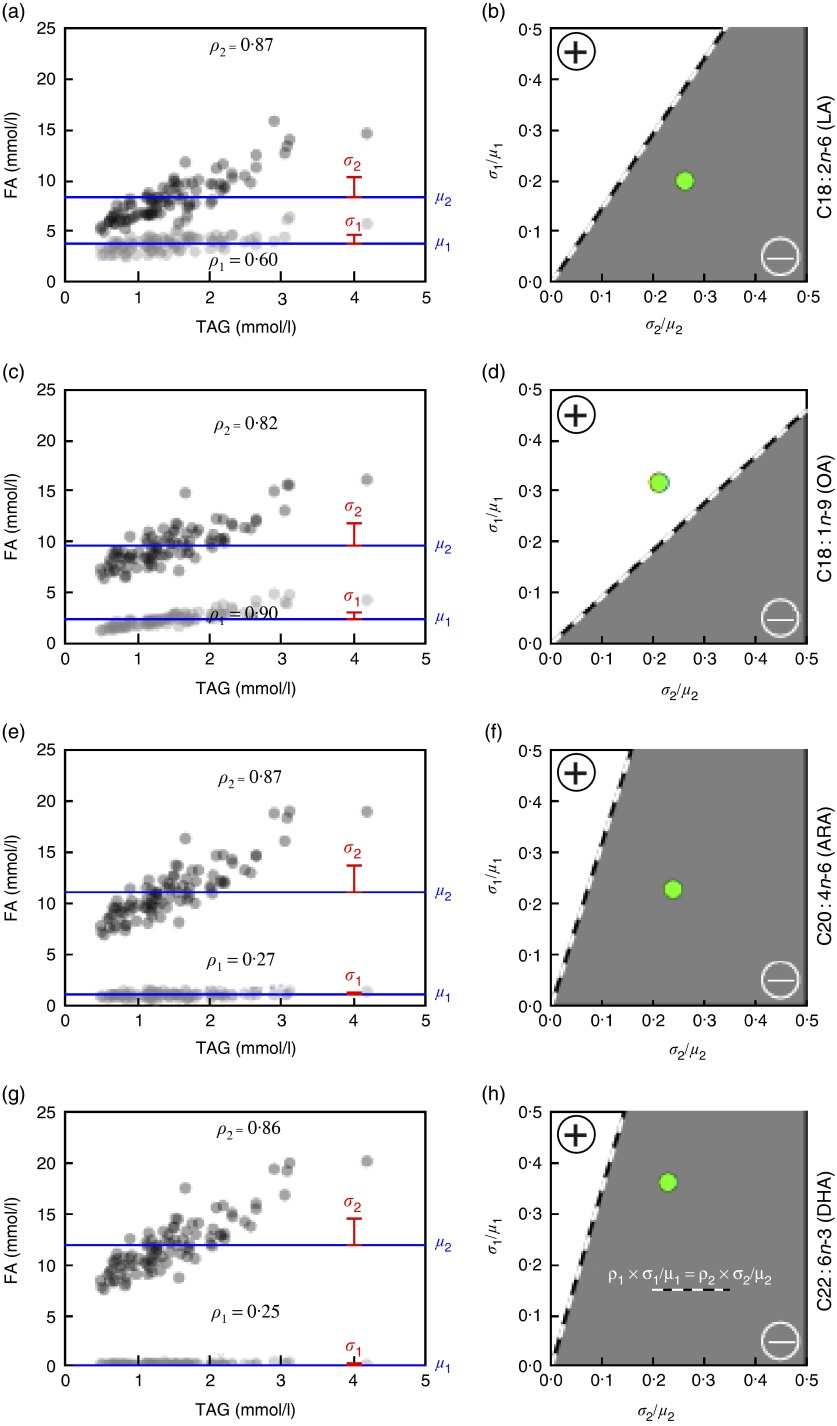
Predicted TAG relationship with (OA, C18 : 1*n*-9), linoleic acid (LA, C18 : 2*n*-6), (ARA, C20 : 4*n*-6) and DHA (C22 : 6*n*-3) based on fatty acid (FA) concentration and variability (Diet, Exercise, Metabolism and Obesity in Older Women (DEMO) cohort). The TAG levels *v*. observed FA concentrations (a, c, e, g), indicating serum concentrations of LA (a, 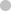), OA (c, 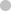), ARA (e, 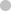) and DHA (g, 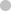), and total FA concentrations without LA, OA, ARA and DHA, respectively (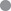). Values are means (*μ*, 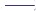) and sd (*σ*, 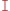), and the sample correlation with TAG (ρ) is shown in the lower (LA, OA, ARA, DHA) or upper (all other FA) part of the respective scatter plots. The correlation between TAG and percentage of total FA will be negative (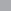, b, d, f, h) if, for a given FA, the product of the correlation (*ρ*_1_) with the TAG levels and its CV (*σ*_1_/*μ*_1_) is smaller than the corresponding product for all other FA concentrations combined (*ρ*_2_× *σ*_2_/*μ*_2_). The percentage of total LA, ARA and DHA expected to be negatively correlated with TAG levels (b, f, h) and the percentage of total OA expected to be positively correlated with TAG levels (d). 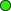, The observed ratios of means and standard deviations.

To better understand the discrepancies observed with different methods of analysis, we derived a mathematical equation that could use the observed concentrations of fatty acids to predict the correlation between TAG and fatty acids when fatty acids were expressed as a percentage of total. As the percentage of total fatty acids is a non-linear function of fatty acid concentrations, we used a first-order Taylor-series expansion to estimate the correlation between TAG and fatty acid expressed as a percentage of total (see the online Supplementary Materials for mathematical details). Importantly, we observed that the variable direction in the relationship only depends on the serum fatty acid concentrations, their statistical variability and the correlation with TAG levels. Mathematically, the sign of the correlation between TAG and percentage of total solely depends on the sign of the term *ρ*_1_ *σ*_1_ *μ*_2_−*ρ*_2_ *σ*_2_ *μ*_1_, where *μ*_1_ denotes the average concentration of a serum fatty acid in the cohort, *μ*_2_ the average of all other fatty acid concentrations, *σ*_1_ and *σ*_2_ the respective standard deviations, and *ρ*_1_ and *ρ*_2_ the respective correlations with the TAG levels (see the online Supplementary Materials for details). Specifically, the correlation between TAG and percentage of total fatty acids will be negative if, for a given fatty acid, the product of the correlation with the TAG levels and its CV is smaller than the corresponding product for all other fatty acid concentrations combined:
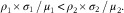



In the case of LA (Fig. 2(a)), the average concentration among the ninety-three samples (DEMO cohort) was 3·83 mmol/l (estimate for *μ*_1_), and the average of all other fatty acids was 8·27 mmol/l (estimate for *μ*_2_). The respective sd were 0·77 mmol/l (estimate for *σ*_1_) and 2·17 mmol/l (estimate for *σ*_2_), yielding estimated CV of 0·20 and 0·26, respectively. The sample correlations with TAG levels were 0·60 (estimate for *ρ*_1_) and 0·87 (estimate for *ρ*_2_), respectively. As 0·12=0·60×0·20<0·87×0·26=0·23, the correlation between TAG and percentage of total LA is predicted to be negative. Fig. 2(b) shows that the observed ratio of means and standard deviations (large green circle) for LA does reside in the predicted (grey) area of negative correlation.

In contrast, the average OA (Fig. 2(c)) concentration among the ninety-three samples was 2·39 mmol/l, and the average of all other fatty acids was 9·70 mmol/l. The respective sd were 0·76 and 2·04 mmol/l, yielding estimated CV of 0·32 and 0·21, respectively; the sample correlations with TAG levels were 0·90 for OA and 0·82 for the sum of all other fatty acids. As 0·90×0·32=0·29>0·17=0·82×0·21, the predicted correlation between TAG and percentage of total OA should be positive (Fig. 2(d)). In addition, the direction of the associations of ARA (Fig. 2(e) and (f)) and DHA (Fig. 2(g) and (h)) with TAG is predicted to be negative as shown. Importantly, comparable directional relationships between these fatty acids and TAG were observed in the replication cohort (online Supplementary Fig. S1).

### Relationship between alternative forms of fatty acid measurement

The mathematical model clearly established that the predicted and observed relationships between TAG and selected fatty acids (LA, OA, ARA and DHA) are in agreement. It was then important to evaluate the relationships between the alternative forms of fatty acid expression (independent of lipid biomarkers) across the wider array of fatty acids assessed in these data. As would be expected, the correlations between total and individual fatty acid concentrations in serum were all positive (not shown). In contrast, the correlation between total fatty acid concentration and the percentage of total fatty acids for an individual fatty acid were observed to be either positive or negative, depending on the fatty acid. Mathematically, the predicted sign of the correlation between total fatty acid concentration and percentage of total fatty acid depends solely on the term *σ*_1_ *μ*_2_(*σ*_1_+*ρ* *σ*_2_)−*σ*_2_ *μ*_1_(*σ*_2_+*ρ* *σ*_1_), whereas, above, *μ*_1_ denotes the average concentration of a serum fatty acid in the population, *μ*_2_ the average of all other fatty acid concentrations, *σ*_1_ and *σ*_2_ the respective standard deviations, and *ρ* the correlation between the serum fatty acid concentration and total fatty acid level less than the fatty acid of interest (see the online Supplementary Materials for details). The correlation between total fatty acid concentration and percentage of total fatty acid will be negative if, for a given fatty acid, its CV is small compared with the coefficient for all other fatty acid concentrations combined, with the exact threshold for the sign depending on *ρ*ː
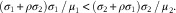



**Fig. 3:**
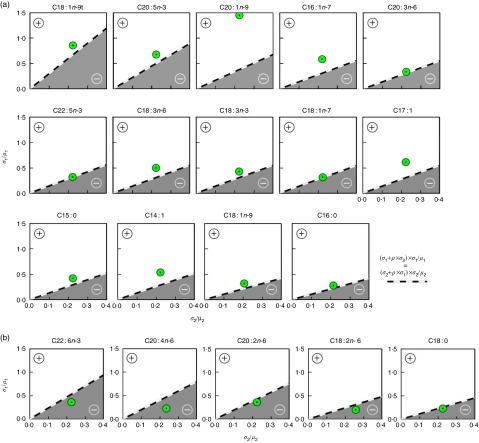
Relationship of fatty acid concentration when reported as a percentage of total and total serum concentration. The expected correlations between percentage of total and total fatty acid concentration as functions of the CV (*σ*_2_/*μ*_2_ x-axis; *σ*_1_/*μ*_1_; y-axis) are shown. The correlation between total fatty acid concentration and percentage of total fatty acid will be negative (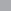) if for a given fatty acid the CV is small compared with the coefficient for all other fatty acid concentrations combined, with the exact threshold for the sign depending on the correlation (*ρ*) between the fatty acid concentration and the sum of the concentrations of all other fatty acids. The sample standard deviations were used to predict the boundaries (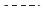) between positive and negative correlations. (a) In all, fourteen fatty acids, including oleic acid, were positively correlated with total fatty acid concentration when expressed as a percentage of total. (b) The five fatty acids, including linoleic acid, negatively correlated with total fatty acid concentration when expressed as a percentage of total. 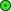, The observed coefficients.

Among the nineteen fatty acids examined, fourteen, including OA, exhibited a positive relationship between the total fatty acid concentration and the individual fatty acid when expressed as percentage of total as evidenced by the location of the observed ratio of fatty acid means and standard deviations (Fig. 3(a); green circle). These include C18 : 1*n*-9*t*, C20 : 5*n*-3, C20 : 1*n*-9, C16 : 1*n*-7, C20 : 3*n*-6, C22 : 5*n*-3, C18 : 3*n*-6, C18 : 3*n*-3, C18 : 1*n*-7, C17 : 1, C15 : 0, C14 : 1, C18 : 1*n*-9 and C16 : 0, in order of increasing value of the correlation coefficient (*ρ*; range 0·32–0·94). In contrast, for five fatty acids, including LA, the correlation between percentage of total for an individual fatty acid and total fatty acid concentration was negative (Fig. 3(b)). For this group of fatty acids (C22 : 6*n*-3, C20 : 4*n*-6, C20 : 2*n*-6, C18 : 2*n*-6 and C18 : 0), the observed ratio of fatty acid means and standard deviations fell in the negative correlation field. Thus, the directionality of the relationship between fatty acids as a percentage of total (a relative expression) and total fatty acid concentration (absolute expression) among fatty acid species is an apparent inherent property and independent of the TAG. Nevertheless, we believed it important to evaluate the impact of the alternative fatty acid expression methods on the relationships between fatty acids and other blood lipids, whose fatty acid content differs qualitatively and quantitatively from that in TAG.

### Relationship between serum fatty acid expression method and other blood lipids

**Fig. 4:**
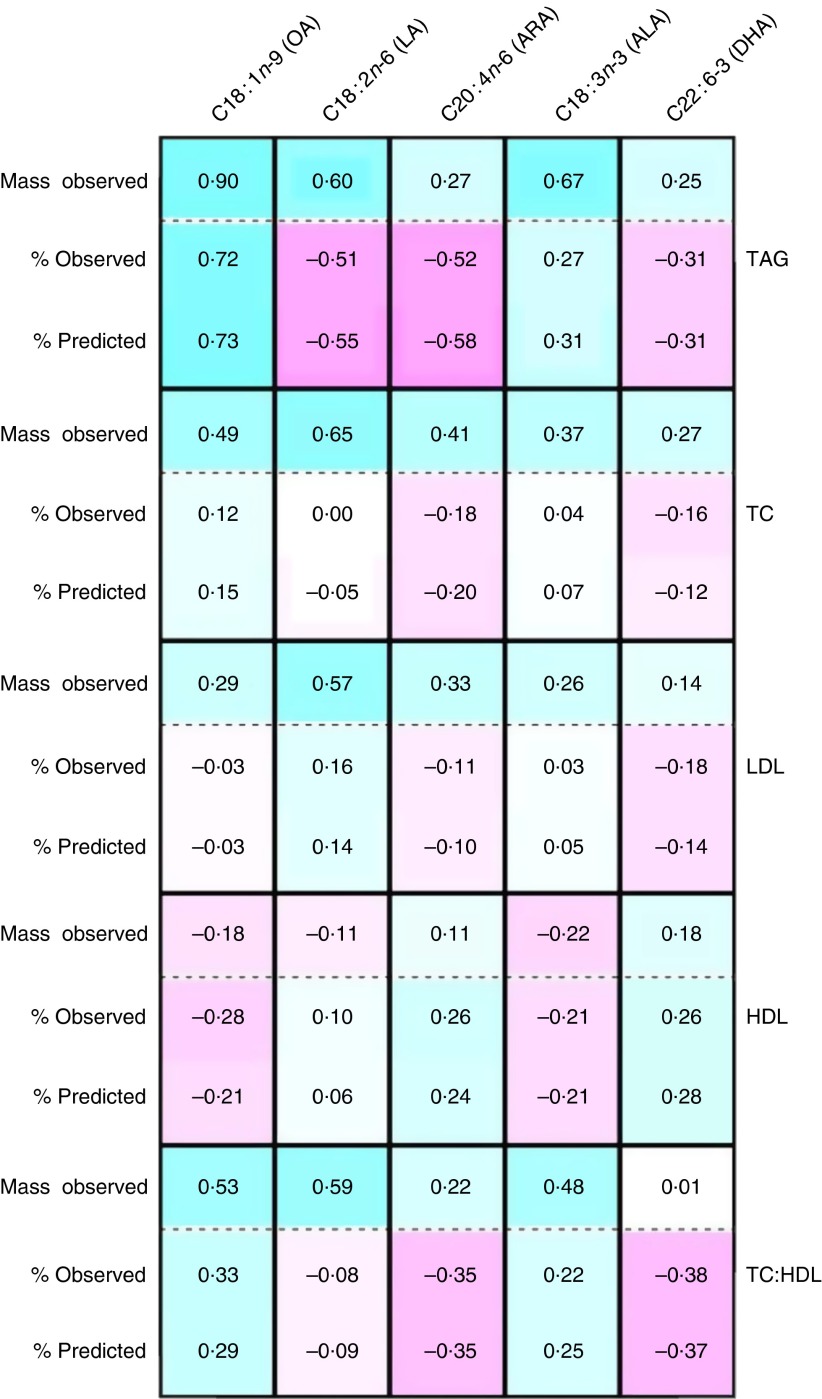
Predicted relationships between blood lipids and selected PUFA (Diet, Exercise, Metabolism and Obesity in Older Women cohort). Sample correlations between cholesterol-containing blood lipids (TAG, total cholesterol (TC), LDL, HDL, ratio of TC:HDL-cholesterol) and selected serum fatty acids (oleic acid (OA), linoleic acid (LA), arachidonic acid (ARA), *α*-linolenic acid (ALA), DHA), using both measured fatty acid concentrations (mg/dl; ‘mass observed’) and percentage of total data (‘% observed’). Also shown are the predicted correlations between blood lipids and percentage of total (‘% predicted’). Positive *v*. negative correlations are highlighted by 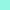 *v*. 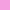 cells, respectively.

In addition to TAG, circulating fatty acids also reside in other circulating complex lipids including LDL, HDL and TC, which have been utilised as disease biomarkers. Fig. 4 summarises the associations between cholesterol-containing blood lipids and selected serum fatty acids (OA, LA, ARA, ALA, DHA) using both concentration (mmol/l) and percentage of total data. With the exception of HDL, the (Pearson’s) correlations of blood lipids with fatty acid concentration (mass) were positive (blue cells), whereas the correlations with percentage of total data were either positive (blue) or negative (pink). Importantly, the mathematically predicted association values for percentage of total were nearly identical (in both magnitude and direction) to those observed for percentage of total fatty acids. As was the case for TAG, the associations of TC, LDL, HDL and the TC:HDL ratio with OA were consistent for both expression methods. Like LA, the long-chain *n*-6 and *n*-3 PUFA (ARA and DHA, respectively) showed a reversal in the direction of associations using the percentage of total fatty acid expression for TAG, TC, LDL and the ratio of TC:HDL-cholesterol. In contrast, associations with HDL tended not to undergo a change in direction between the fatty acid expression methods, except for LA, for which the associations were not very robust. The associations between TAG and DHA were comparable (for mass observed, positive; percentage of total observed and predicted, negative) to that for the association between TAG and EPA+DHA (mass observed, 0·30; percentage of total observed, −0·21, and predicted, −0·19). Nearly identical results, both in the robustness and direction of correlations, were observed in the replication data set (online Supplementary Fig. S2). Overall, the mathematically modelling technique appears to be useful for predicting a reversal in the direction on associations between fatty acid expression methods and blood lipids.

## Discussion

The identification of relationships between fatty acids levels and disease biomarkers are important to understand potential roles of fatty acids in human disease as well as to predict disease risk or monitor disease progression. Consequently, numerous studies over the past 50 years have examined the relationships between dietary intake of fatty acids and circulating and cellular levels of fatty acid and disease biomarkers, disease incidence and progression^(^ ^22^ ^,^ ^23^ ^,^ ^34^ ^–^ ^40^ ^)^. Importantly, these studies have also been the basis of dietary fatty acid recommendations for human populations^(^ ^41^ ^,^ ^42^ ^)^.

However, numerous factors complicate the assumption that there is a link between fatty acid intake levels with disease risk or to develop dietary recommendations. For example, multiple pools of fatty acids, including both dietary fatty acids as well as endogenously synthesised fatty acids from tissues such as the liver and adipose, contribute to circulating fatty acid levels. In addition, adipose tissue is a major storage repository for fatty acids, which can be released into circulation under a variety of conditions. Moreover, recent studies show that there can be major differences in the frequencies of genetic variants that impact fatty acid levels and particularly conversion of *n*-6 and *n*-3, eighteen-carbon PUFA to long-chain highly unsaturated fatty acids in different human populations^(^ ^12^ ^,^ ^13^ ^,^ ^43^ ^,^ ^44^ ^)^. All of these are key factors that contribute to circulating and cellular levels of fatty acids and complicate studies that attempt to associate dietary fatty acids and disease biomarkers or risk.

In addition, fatty acids are measured in a wide variety of circulating (plasma, serum, plasma phospholipids and cholesterol esters) and cellular compartments (erythrocytes or leucocytes) or mixtures (whole blood) often without a great deal of consideration for why a particular compartment was used. Total circulating fatty acids represent a combination of free fatty acids and those in complex lipids (esterified to TAG, cholesterol esters and phospholipids) in lipoprotein particles. Each of these compartments contains different fatty acid profiles^(^ ^45^ ^,^ ^46^ ^)^. For example, Edelstein^(^ ^45^ ^)^ showed that, in human serum, *n*-6 PUFA account for 22 % of the fatty acids in the TAG, 38 % in the phospholipids and 60 % in the cholesterol esters. In contrast, *n*-3 PUFA accounts for only 1·8 % of the fatty acids in the TAG, 3·5 % in the phospholipids and 1·7 % in the cholesterol esters. This issue is made even more complex given that there are hundreds of fatty acid-containing molecular species contained within glycerolipid, phospholipid and sphingolipid classes in human plasma^(^ ^47^ ^)^. Therefore, results garnered from any study depend on which blood fraction or fractions are examined. Consequently, to avoid confusion and discrepancies, it is important to consider the specific hypothesis that is being addressed before determining the compartment that is to be measured. For example, there are circumstances where measurements of free fatty acids or particular complex lipid (phospholipid, glycerolipid or cholesterol ester) classes or molecular species are necessary to answer a specific question. In other circumstances, it is better to measure the plasma (serum) compartment as a whole given that specific fatty acids are selectively distributed in certain lipid classes and molecular species.

As pointed out in this study, another potential complicating factor in analysing both circulating and cellular fatty acids is the way in which fatty acid data are expressed. This latter concern has been a subject of debate for over 20 years^(^ ^40^ ^,^ ^48^ ^,^ ^49^ ^)^. Fatty acids have been historically expressed as a percentage of total fatty acids in a given compartment. Although this is a convenient and seemingly straightforward method of data presentation, there are mathematical considerations that must be taken into account that can impact the interpretation of the data garnered from such an analysis. The key finding of this study is that the manner in which fatty acid data were expressed (percentage of total *v*. fatty acid concentrations) has a tremendous impact on the relationship between circulating fatty acids and circulating blood lipids.

This was particularly true for *n*-6 PUFA. The literature suggests that serum LA is inversely associated with TAG, TC and LDL-cholesterol^(^ ^50^ ^–^ ^52^ ^)^ when fatty acid data are expressed as a percentage of total. The percentage of total data from the DEMO cohort confirms this inverse relationship between percentage of total serum LA (Fig. 1 and 2) and TAG, but the direction of this relationship reversed when LA levels were expressed as a concentration of the fatty acid in serum (mmol/l). Similarly, inverse associations have been reported between ARA and TAG or LDL-cholesterol^(^ ^50^ ^,^ ^52^ ^)^, but again these are reversed when ARA is expressed as concentration. We caution though that conclusions can be drawn only for fatty acids measured here and their respective ranges of concentrations observed, as other relationships might exist, for example, among much higher circulating levels of DHA, or among other fatty acids.

On the basis of the derivations and findings reported in this manuscript, we also recommend that previously reached conclusions about LA and AA and their relationship to lipid profile and CHD should be re-visited. The issue of whether relationships reported using absolute *v*. relative concentrations agree or disagree is purely mathematical, and depends on the mean levels of these concentrations and their respective covariances. This paper reveals that the different methods of expressing concentrations lead to dissimilar correlations between blood lipids and some fatty acids, a phenomenon that might well be present among other biomarker relationships as well.

It is not surprising that there are positive associations between total fatty acids and individual fatty acids when expressed as concentration. It is also expected that total fatty acid concentrations would be associated with TC, LDL-cholesterol and TAG as fatty acids are esterified to cholesterol, TAG and phospholipids in circulation. These complex lipids are packaged within lipoprotein particles as major transporters of fatty acids to cells and tissues. However, in an attempt to standardise and simplify the data by using a percentage of total analysis, it does appear that there is a great potential for misinterpreting such data, especially in light of the number of highly influential papers and a recent meta-analysis that have concluded that certain fatty acids, and particularly *n*-6 PUFA, have cardio-protective attributes^(^ ^37^ ^,^ ^41^ ^)^.

It could be argued that separating circulating fatty acids into fractions such as plasma phospholipids is a better approach and the percentage of total data would be more reliable in this context. However, this would again assume that the size of the fraction is uniform in all individuals and/or that all individual fatty acids within the fraction increased or decreased at the same rate. Taken together, these data suggest that when examining fatty acids in total serum (or plasma) or in isolated fractions such as plasma phospholipids the concentration of individual fatty acids should be measured. This method of analysis is particularly important when attempting to determine the relationships between fatty acids and disease biomarkers.

### Conclusion

Associations between circulating fatty acids and blood lipids have influenced our view of fatty acid with regard to their importance in human disease and dietary recommendations. However, we show that different methods of fatty acid expression result in non-uniform relationships between certain circulating fatty acids and circulating complex lipids such as TAG, and LDL, HDL or TC. In addition, we have demonstrated that the commonly used percentage of total expression method can be mathematically modelled using fatty acid concentration data, and this provides a means of predicating a reversal in the direction of association. Thus, the method by which circulating and potentially cellular fatty acids are expressed appears to be important when interpreting relationships between fatty acid levels and blood lipid levels. These data suggest that relationships between fatty acids and other biomarkers and indices of CHD should be re-visited with this in mind.

## Supplementary material

For supplementary material/s referred to in this article, please visit http://dx.doi.org/doi:10.1017/S0007114515004341
